# Isolation of aerobic cultivable cellulolytic bacteria from different regions of the gastrointestinal tract of giant land snail *Achatina fulica*

**DOI:** 10.3389/fmicb.2015.00860

**Published:** 2015-08-20

**Authors:** Guilherme L. Pinheiro, Raquel F. Correa, Raquel S. Cunha, Alexander M. Cardoso, Catia Chaia, Maysa M. Clementino, Eloi S. Garcia, Wanderley de Souza, Susana Frasés

**Affiliations:** ^1^Diretoria de Metrologia Aplicada às Ciências da Vida, Instituto Nacional de Metrologia, Qualidade e TecnologiaRio de Janeiro, Brazil; ^2^Laboratório de Ultraestrutura Celular Hertha Meyer, Instituto de Biofísica Carlos Chagas Filho, Universidade Federal do Rio de JaneiroRio de Janeiro, Brazil; ^3^Centro Universitário Estadual da Zona Oeste, Unidade Universitária de BiologiaRio de Janeiro, Brazil; ^4^Departamento de Microbiologia, Instituto Nacional de Controle da Qualidade em Saúde, Fundação Oswaldo CruzRio de Janeiro, Brazil; ^5^Departamento de Bioquímica e Biologia Molecular, Instituto Oswaldo Cruz, Fundação Oswaldo CruzRio de Janeiro, Brazil

**Keywords:** *Achatina fulica*, cellulolytic bacteria, carboxymethycellulose, microbial diversity

## Abstract

The enzymatic hydrolysis of cellulose by cellulases is one of the major limiting steps in the conversion of lignocellulosic biomass to yield bioethanol. To overcome this hindrance, significant efforts are underway to identify novel cellulases. The snail *Achatina fulica* is a gastropod with high cellulolytic activity, mainly due to the abundance of glycoside hydrolases produced by both the animal and its resident microbiota. In this study, we partially assessed the cellulolytic aerobic bacterial diversity inside the gastrointestinal tract of *A. fulica* by culture-dependent methods and evaluated the hydrolytic repertoire of the isolates. Forty bacterial isolates were recovered from distinct segments of the snail gut and identified to the genus level by 16S rRNA gene sequence analysis. Additional phenotypic characterization was performed using biochemical tests provided by the Vitek2 identification system. The overall enzymatic repertoire of the isolated strains was investigated by enzymatic plate assays, containing the following substrates: powdered sugarcane bagasse, carboxymethylcellulose (CMC), p-nitrophenyl-β-D-glucopyranoside (pNPG), p-nitrophenyl-β-D-cellobioside (pNPC), 4-methylumbelliferyl-β-D-glucopyranoside (MUG), 4-methylumbelliferyl-β-D-cellobioside (MUC), and 4-methylumbelliferyl-β-D-xylopyranoside (MUX). Our results indicate that the snail *A. fulica* is an attractive source of cultivable bacteria that showed to be valuable resources for the production of different types of biomass-degrading enzymes.

## Introduction

Cellulolytic organisms are ubiquitous in nature. Both fungi and bacteria have been heavily exploited for their abilities to produce a wide variety of cellulases and hemicellulases. Traditionally, significant emphasis has been placed on the use of fungi because they produce high amounts of extracellular enzymes, which can be easily purified and used as commercial cellulase cocktails (Gusakov and Sinitsyn, 2012). However, novel glycoside hydrolases from bacteria have been isolated and characterized in the last few years. Bacteria have some advantages over fungi in certain aspects. In particular, they usually have a higher growth rate allowing for faster production of recombinant enzymes (Maki et al., 2009). In addition, some glycoside hydrolases from bacteria are assembled in multi-enzyme complexes that provide increased synergy, stability, and catalytic efficiency (Hou et al., 2006; Jiang et al., 2006; Waeonukul et al., 2009), while others display modular architecture (Cann et al., 1999; Zhang et al., 2014) or are multifunctional, harboring both endoglucanase and xylanase activities in the same polypeptide (Pérez-Avalos et al., 2008). Finally, cellulolytic bacteria have been isolate from harsh climate conditions (Soares et al., 2012). As consequence, their enzymes are more stable under extreme conditions (high temperature, extremes of pH) that may occur during bioconvertion processes, and this may increase the overall efficiency of the enzymatic hydrolysis and fermentation (Maki et al., 2009).

Over the years, culturable cellulolytic bacteria have been isolated from a wide variety of environments such as compost piles, decaying plant material originating from agricultural wastes, feces of ruminants, soil, gastrointestinal tract of insects, and from extreme environments such as hot springs (Doi, 2008). Screening for cellulase producing organisms may be accomplished through medium enrichment with crystalline cellulose, followed by 16S rRNA sequencing to determine the composition of the bacterial communities present and evaluate whether families containing cellulolytic species are present. Strains with cellulolytic potential can be isolated by subsequent subcultures in the enriched culture medium containing cellulose as carbon source (Maki et al., 2009; Rastogi et al., 2009). Alternatively, screening of cellulases produced by bacterial isolates may be accomplished by their cultivation in solid media containing carboxymethylcellulose (CMC) as sole carbon source, followed by Congo Red staining (Hankin and Anagnostakis, 1977). CMC is a highly specific substrate for endo-acting cellulases, as its structure has been engineered to decrystallize cellulose and create amorphous sites that are ideal for endoglucanase action, called CMCase, that cleaves intramolecular β-1,4-glucosidic bonds randomly, resulting in a dramatic reduction of the degree of polymerization and specific viscosity of CMC (Zhang et al., 2006). Although CMC has become a commonly used surrogate for cellulose, as many had associated whole cellulase activity with CMC hydrolysis (Liang et al., 2014), cellobiohydrolases are shown to be dominant in the degradation of crystalline (e.g., Avicel) and not soluble (e.g., CMC) cellulose (Zhang et al., 2006).

The giant land snail *Achatina fulica* is a terrestrial pulmonate gastropod mollusk native to East Africa that is considered an invasive pest in most of the territories in which it was introduced by human intervention. Due to its voracious appetite, great environmental adaptability, high growing and reproductive rates, this mollusk is now considered to be the most destructive terrestrial gastropod worldwide, causing ecological disequilibrium and agricultural losses (Albuquerque et al., 2008; Thiengo et al., 2008). Its success as an invasive species is mainly due to its ability to process a broad variety of vegetable organic matter. In addition to their own enzymatic repertoire, land snails contain an intriguing and adaptable microbiota that promotes the fast hydrolysis of lignocellulosic plant biomass, contributing to their impressive digestive efficiency (60–80%) (Charrier and Daguzan, 1980; Cardoso et al., 2012a). Also, recent metagenomic analysis of the crop microbiota of this snail revealed an abundance of sequences coding for oligosaccharide-degrading enzymes (36%) as well as many novel cellulose and hemicellulase coding sequences (Cardoso et al., 2012b). Although the resident bacterial diversity of *A. fulica* has been investigated recently using culture-independent molecular analysis (Pawar et al., 2012; Cardoso et al., 2012a), the cellulolytic capacity of the described bacterial communities was not assessed. Furthermore, cultivable bacteria diversity within pulmonate land snails has been partially investigated in *Helix pomatia* and *Cornu aspersum* (Charrier et al., 2006), but not been assessed in *A. fulica*. Thus, there is still need for a detailed study on the microflora from this land snail in order to identify specific bacterial isolates that are directly involved with the lignocellulosic biomass degradation.

The main focus of this work was to isolate cultivable CMC-degrading bacteria from the digestive tract of *A. fulica* in order to evaluate the biotechnological potential for their secreted hydrolytic enzymes. We were able to obtain 40 bacterial isolates, which were identified by 16S rRNA gene sequencing and additionally evaluated by phenotypic characterization using biochemical markers. The hydrolytic repertoire of the strains was investigated by enzymatic plate assays, using distinct substrates. This is the first study that focused on the evaluation of cellulolytic bacterial communities resident in *A. fulica*, showing that this land snail is a valuable source of bacterial species that can be cultivated to produce different types of cellulases.

## Materials and methods

### Sampling

Three field-collected *A. fulica* snails weighing in the range of 70–80 g were captured in Rio de Janeiro, Brazil. To minimize the occurrence of transient bacteria within digestives fluids, the snails were kept inside plastic boxes (40 cm long, 20 cm wide, 20 cm high) under starvation conditions, without water and or other substrates for 24 h after capture before sample collection.

### Recovery of bacteria from crop, intestine, and rectum luminal fluids

The snails were anesthetized according to Chung (1985), by injecting the pallial cavity with 0.5 mL of 0.01% succinylcholine chloride in 2% MgCl_2_ solution and immediately dissected inside a biosafety cabinet. Digestive tubes were placed in a sterile Petri dish covered with a wax layer and three segments were isolated per snail: the first digestive cavity, the crop (C), that contains great amounts of a red viscous digestive fluids; the intestine (I), which comprises the proximal intestine (PI), embebed within the digestive gland and the uncovered distal intestine (DI); and the rectum (R), which is the last digestive section (Charrier and Brune, 2003). The selected segments were opened using a sterile blade and luminal contents from the same segment were pooled and suspended vigorously in a 15-ml Falcon tube containing 10 ml of PBS (phosphate-buffered saline) (8 g.l^−1^ NaCl; 0.2 g.l^−1^ KCl; 1.44 g.l^−1^ Na_2_HPO_4_·12H_2_O; 0.24 g.l^−1^ KH_2_PO_4_; pH 7.6). Then, the suspension was centrifuged at 5000 × g for 15 min. Supernatant was discarded to remove endogenous cellulases and the pellet was washed twice in 10 mL of sterile PBS. Finally, pellets were suspended in 1 ml of sterile PBS.

#### Endoglucanase activity as first selection pressure (CMCase activity)

The three luminal suspensions (crop, intestine, and rectum) were serially diluted in PBS, ranging from 10^−3^ to 10^−5^, and plated in triplicate onto solid minimal media (MM) containing carboxymethylcellulose (CMC) (carboxymethylcellulose sodium salt, low viscosity, from Sigma Aldrich) as the sole carbon source [CMC media: 5 g.l^−1^ CMC; 20 g.l^−1^agar; 6.8 g.l^−1^ Na_2_HPO_4_; 3 g.l^−1^KH_2_PO_4_; 0.5 g.l^−1^ NaCl; 1.3 g.l^−1^ (NH_4_)_2_SO_4_ and 0.5 g.l^−1^ MgSO_4_.7H_2_O]. Plates were incubated for 3 days at 30°C and the resulting discrete colonies were picked and streaked four times onto new CMC plates to insure they could utilize CMC as the sole carbon source and were not using residual nutrients from the intestinal fluids (Robson and Chambliss, 1989). Pure isolates were subjected to Congo red staining (Teather and Wood, 1982). Strains were designated C to indicate isolation from the crop; I, from intestine; R, from rectum. The use of Congo-Red as an indicator for CMC degradation in an agar medium provides the basis for a rapid and sensitive first screening test for cellulolytic microbes. Isolates were maintained on CMC plates for additional experiments and also stored in 15% glycerol at −80°C for future use. After this first screening, isolates were analyzed for their capacity to degrade other polysaccharides.

#### Enzymatic plate assay

In order to evaluate the repertoire of secreted hydrolytic enzymes, the isolates were grown on MM plates containing distinct substrates: 1 mM pNPC (p-nitrophenyl-β-D-cellobioside) (Deshpande et al., 1984); 0.04% MUC (4-methylumbelliferyl-β-D-cellobioside) (Heptinstall et al., 1986); 0.04% MUG (4-methylumbelliferyl-β-D-glucopyranoside) (Heptinstall et al., 1986); 1 mM pNPG (p-nitrophenyl-β-D-glucopyranoside) (Deshpande et al., 1984); 0.04% MUX (4-methylumbelliferyl-β-D-xylopyranoside) (Bruyne and Loontiens, 1965) or 0.5% powdered sugarcane bagasse (Lucena et al., 2011) as carbon sources. Plates were incubated at 30°C for 3 days before enzyme detection.

#### Visualization of enzymatic activity

For bagasse and CMC substrates, enzyme detection was based on the appearance of a clearance halo surrounding the colonies after Congo red staining (Robson and Chambliss, 1989). Colonies harboring negative halos up to 2 mm wide relative to the colony boundary were classified as positive (+) for the CMCase secretion and those that showed halos greater than 2 mm were classified as double positive (++). For the fluorescent substrates MUC, MUG, and MUX, plates were examined under UV light (302 nm) on a Gel Doc XR+ Imaging System (Bio-Rad, Hercules, USA). Pictures were taken with the Image Lab 2.0 Software (Bio-Rad) using the automatic exposure time mode. Colonies harboring fluorescent halos were classified as positive (+) when the halos could only be visualized using the exposure time optimized for faint bands (high exposure time). Colonies whose fluorescent halos were detected upon intense bands exposure time mode (low exposure time) were categorized as double positive (++) for the respective enzyme secretion. For the colorimetric substrates pNPC and pNPG, the enzyme secretion was proportional to the development of yellow colored halos surrounding the colonies. Colonies harboring colored halos up to 5 mm wide relative to the colony boundary were classified as positive (+) and those who showed halos greater than 5 mm were classified as double positive (++) for the enzyme secretion.

### Phenotypic bacterial characterization

For preliminary morphological characterization isolates Gram stain was performed and evaluated by light microscopy. Cultures were grown in liquid CMC medium at 30°C for 2 days at 150 rpm. After, cells were washed with sterile PBS and 5 uL of the suspension were transferred to glass slides, heat-fixed and stained according to Gram's procedure (Holt et al., 1994). The stained slides were imaged under a Leica DM 5000B microscope (Leica Microsystems, Buffalo Grove, IL, USA). Pictures were taken at 100X magnification using Leica Application Suite Software (Leica Microssystems).

#### Biochemical characterization

Bacterial isolates were analyzed by biochemical tests measuring carbon source utilization, enzymatic activities, and antibiotic resistance using Vitek2 identification System (BioMérieux, Marcy l'Étoile, France), according to the manufacturer's recommendations. Briefly, bacterial suspensions turbidity was adjusted to 0.5 McFarland standard in 0.45% sodium chloride. Then, GN (Gram negative), GP (Gram positive) cards, and bacterial suspensions were manually loaded into the Vitek2 System.

The GN identification card includes tests for the following reactions: beta-galactosidase, beta-N-acetyl-glucosaminidase, glutamyl-arylamidase-pNAl, gamma-glutamyl-transferase, beta-glucosidase, beta-xylosidase, beta-alanine-arylamidase-pNA, alpha-glucosidase, beta-N-acetyl-galactosaminidase, alpha-galactosidase, phosphatase, glycine-arylamidase, beta-glucuronidase, glu-gly-arg-arylamidase, ala-phe-pro-arylamidase, L-pyrrolydonyl-arylamidase, L-proline-arylamidase, lípase, tyrosine-arylamidase, urease, ornithine-decarboxylase, lysine-decarboxylase, fermentation of glucose, H2S-production, and Ellman's test. The GN card also tests acid production from the following substrates: sucrose, glucose, adonitol, arabitol, cellobiose, maltose, mannitol, mannose, palatinose, sorbitol, trehalose, and tagatose. Finally, the following tests are also included: assimilation of malate, lactate, citrate, malonate, 5-keto-D-gluconate, coumarate, and histidine, as well as alkalinization of succinate and lactate.

The GP identification card includes test for the following reactions: phosphatidylinositol phospholipase C, arginine dihydrolase (two tests), β-galactosidase, α-glucosidase, alanine-phenylalanine-proline arylamidase, L-aspartate arylamidase, β-galactosidase, α-mannosidase, alkaline phosphatase, l-leucine arylamidase, proline arylamidase, β-glucuronidase (two tests), α-galactosidase, L-pyrrolidonyl-arylamidase, alanine arylamidase, tyrosine arylamidase and urease. The GP identification card also tests acid production from the following substrates: amygdalin, xylose, α-cyclodextrin, sorbitol, galactose, ribose, lactate, lactose, N-acetyl-glucosamine, maltose, mannitol, mannose, methyl-β-d-glucopyranoside, pullulan, raffinose, salicin, sucrose, and trehalose. Finally, growth in 6.5% NaCl as well as tests for resistance to polymyxin B, bacitracin, novobiocin, O129, and optochin are also included in the GP identification card.

#### 16S rRNA gene cloning and sequencing

Bacterial genomic DNA was extracted using the “GenElute Bacterial Genomic DNA” kit (Sigma-Aldrich; St. Louis, USA) according to manufacturer's instructions and PCR-amplified with the universal bacterial primers 27BF (5′-*AGAGTTTGATCCTGGCTCAG*-3′) and 907RAB (5′-*TTTGAGTTTMCTTAACTGCC*-3′) for the 16S rRNA gene (Weisburg et al., 1991), using the following conditions: 5 min hot start at 94°C, followed by denaturation for 60 s at 94°C, annealing for 30 s at 54°C and 60 s of extension at 72°C. On the 35th and final cycle, the extension time was increased to 7 min. PCR products were purified using the “QIAquick PCR purification Kit” (Qiagen; Hilden, Germany) following the manufacturer's instructions. Sequencing was bidirectional (primers 27BF and 907RAB) and was performed using the MegaBace1000 DNA analysis system (GE Healthcare; Buckinghamshire, UK). Partial 16S rRNA sequences generated in this study have been deposited in GenBank under the accession numbers sequentially numbered from KF530754 (C1) to KF530793 (R40.2). Supplementary Table 1S lists the correspondence of isolate ID to Genbank accession.

#### Bioinformatic analysis

Sequence assemblies were obtained with the CAP3 Assembly Program (http://pbil.univ-lyon1.fr/cap3.php) and searches against GenBank non-redundant databases were performed using the Basic Local Alignment Search Tool (BLAST) algorithm (Altschul et al., 1990). Alignments with representative bacterial sequences obtained at GenBank databases were carried out using MUSCLE software (Edgar, 2004). Phylogenetic analyses were carried out with MEGA software (Tamura et al., 2011) and the tree was constructed by neighbor-joining algorithm based on distance estimates calculated by the Kimura-2 parameter which includes a bootstrap test with 1000 replicates.

## Results

### Isolation of cellulolytic aerobic bacteria from crop, intestine, and rectum luminal fluids from the giant snail *Achatina Fulica*

**Figure 1:**
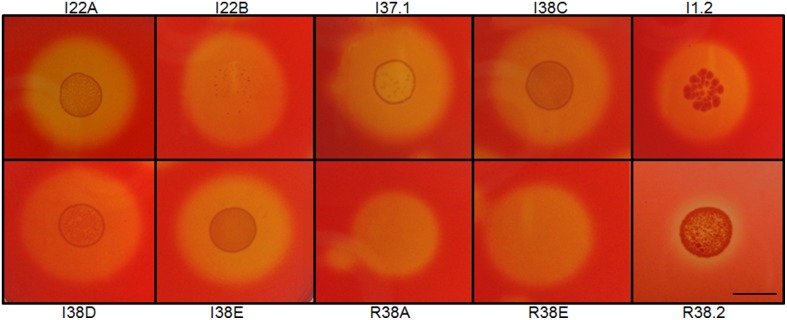
**Examples of Congo red staining of cellulolytic aerobic bacteria from the gastrointestinal lumen of the giant snail ***Achatina fulica*****. Strains were designated as C to indicate isolation from the crop; I, from intestine; R, from rectum. Numerals indicated clone number. Scale bar, 1.0 cm.

In this work, we investigated the composition of the cultivable CMC-degrading bacterial community in three parts of the digestive tract of the land snail *A. fulica*: crop (C), intestine (I), and the rectum (R). In our screening, a total of 40 CMC-degrading isolates were obtained from all combined tested snail samples. For the preliminary evaluation of the cellulolytic activity, we performed the Congo red staining method on CMC agar plates to identify the CMCase-secreting isolates (Figure 1). Of the 40 isolates able to grown on CMC plates, a total of 24 bacterial isolates hydrolyzed CMC. Sixteen isolates were able to grew on CMC as their sole carbon sources, but did not display visible degradation halos for CMCase (**Table 4**).

### Characterization of bacterial isolates by phylogenetic and enzymatic analysis

The DNA fragments for 16S rRNA genes of the 40 isolates were amplified and sequenced. The resulting sequences were subjected to Blast analysis against GenBank non-redundant databases. The Blast matching with the highest score is shown for each isolate (Table 1). Our 40 isolates belonged to three bacterial phyla, namely *Proteobacteria* (24 isolates), *Actinobacteria* (13 isolates), and *Firmicutes* (3 isolates). These 40 isolates showed their closest matches to 13 distinct genera, 6 of the *Proteobacteria* phyla (*Aeromonas, Pseudomonas, Klebsiella, Enterobacter, Stenotrophomonas*, and *Paracoccus*), 5 of *Actinobacteria* (*Streptomyces, Cellulosimicrobium, Agromyces, Microbacterium*, and *Nocardiopsis*), and 2 of *Bacteroidetes* (*Sphingobacterium* and *Flavobacterium*). In the crop, members of the genera *Aeromonas* were the most predominant and accounted for 38% of total numbers of isolates identified. In intestine, species of the genera *Cellulosimicrobium* spp. were the most predominant (60% of total intestine isolates). Interestingly, all of the representatives of the *Aeromonas* genera identified in our study were recovered exclusively from the crop fluids, whereas species of *Cellulosimicrobium* were recovered only from intestine. In the rectum, there was not a predominance of any cellulolytic isolate over the others.

**Table 1:** **Molecular identification of the isolates**.

**Isolate**	**16S identification**	**Gram**	**Accession**	**Genbank description**	**Score**	**Coverage (%)**	**Identity (%)**	**Phylum**
C1	*Pseudomonas*	−	AB681730.1	*Pseudomonas nitroreducens* subsp. *thermotolerans* gene for 16S rRNA, partial cds.	1487	99	99	*Proteobacteria*
C2	*Klebsiella*	−	AB680060.1	*Klebsiella pneumoniae* gene for 16S rRNA, partial sequence, strain: NBRC 3318	1395	100	98	*Proteobacteria*
C3	*Sphingobacterium*	−	NR_042134.1	*Sphingobacterium mizutaii* strain DSM 11724 16S ribosomal RNA, partial sequence	1014	100	99	*Bacteroidetes*
C5	*Paracoccus*	−	AB680283.1	*Paracoccus denitrificans* gene for 16S rRNA, partial sequence, strain: NBRC 12442	1371	99	100	*Proteobacteria*
C6	*Paracoccus*	−	JQ321836.1	*Paracoccus* sp. YF1 16S ribosomal RNA gene, partial sequence	1266	99	100	*Proteobacteria*
C7	*Sphingobacterium*	−	FJ459994.1	*Sphingobacterium multivorum* 16S ribosomal RNA gene, partial sequence	1485	99	99	*Bacteroidetes*
C8	*Microbacterium*	+	AB646581.2	*Microbacterium* sp. SL10 gene for 16S ribosomal RNA, partial sequence	1256	99	96	*Actinobacteria*
C9	*Pseudomonas*	−	AB646255.1	*Pseudomonas* sp. H-8-1-3 gene for 16S ribosomal RNA, partial sequence	1500	100	99	*Proteobacteria*
C10	*Flavobacterium*	−	DQ168834.1	Uncultured *Flavobacterium* sp. clone J16 16S ribosomal RNA gene, partial sequence	1432	100	98	*Bacteroidetes*
C11	*Aeromonas*	−	NR_029252.1	*Aeromonas punctata* strain ATCC 15468 16S ribosomal RNA, partial sequence	1515	100	99	*Proteobacteria*
C12	*Aeromonas*	−	NR_029252.1	*Aeromonas punctata* strain ATCC 15468 16S ribosomal RNA, partial sequence	1391	100	96	*Proteobacteria*
C13.4	*Aeromonas*	−	NR_029252.1	*Aeromonas punctata* strain ATCC 15468 16S ribosomal RNA, partial sequence	1330	100	98	*Proteobacteria*
C14	*Pseudomonas*	−	GU979230.1	*Pseudomonas* sp. WP6 16S ribosomal RNA gene, partial sequence	1426	100	99	*Proteobacteria*
C15	*Aeromonas*	−	NR_029252.1	*Aeromonas punctata* strain ATCC 15468 16S ribosomal RNA, partial sequence	1068	96	97	*Proteobacteria*
C16	*Microbacterium*	+	AB646581.2	*Microbacterium* sp. SL10 gene for 16S ribosomal RNA, partial sequence	1482	100	98	*Actinobacteria*
C18	*Pseudomonas*	−	JQ701740.1	*Pseudomonas putida* strain jvu23 16S ribosomal RNA gene, partial sequence	1423	99	99	*Proteobacteria*
C19	*Aeromonas*	−	NR_029252.1	*Aeromonas punctata* strain ATCC 15468 16S ribosomal RNA, partial sequence	1465	98	98	*Proteobacteria*
C20	*Pseudomonas*	−	AB513735.1	*Pseudomonas putida* gene for 16S ribosomal RNA, partial sequence, strain: 1106	1439	99	98	*Proteobacteria*
C21.1	*Aeromonas*	−	NR_029252.1	*Aeromonas punctata* strain ATCC 15468 16S ribosomal RNA, partial sequence	1461	99	99	*Proteobacteria*
C22	*Aeromonas*	−	NR_029252.1	*Aeromonas punctata* strain ATCC 15468 16S ribosomal RNA, partial sequence	1504	99	99	*Proteobacteria*
C23	*Klebsiella*	−	NR_025635.1	*Klebsiella variicola* strain F2R9 16S ribosomal RNA, partial sequence	1450	99	98	*Proteobacteria*
C24.1	*Aeromonas*	−	AB626132.1	*Aeromonas* caviae gene for 16S rRNA, partial sequence, strain: JCM 1060	1456	100	98	*Proteobacteria*
C24.2	*Paracoccus*	−	NR_026457.1	*Paracoccus pantotrophus* strain ATCC 35512 16S ribosomal RNA, partial sequence	976	98	99	*Proteobacteria*
C25	*Aeromonas*	−	JF920485.1	*Aeromonas caviae* strain E4EL26 16S ribosomal RNA gene, partial sequence	1506	98	99	*Proteobacteria*
I 1.2	*Streptomyces*	+	NR_043823.1	*Streptomyces kunmingensis* strain NRRL B-16240 16S ribosomal RNA, partial sequence	1450	100	99	*Actinobacteria*
I22A	*Cellulosimicrobium*	+	AB188217.1	*Cellulosimicrobium* sp. TUT1222 gene for 16S rRNA, partial sequence	1421	100	99	*Actinobacteria*
I22B	*Cellulosimicrobium*	+	JQ659848.1	*Cellulosimicrobium funkei* strain R6-417 16S ribosomal RNA gene, partial sequence	1480	99	99	*Actinobacteria*
I28A	*Klebsiella*	−	AB114637.1	*Klebsiella* sp. PN2 gene for 16S rRNA	1443	97	99	*Proteobacteria*
I32.1	*Enterobacter*	−	JQ396391.1	*Enterobacter* sp. PXG11 16S ribosomal RNA gene, partial sequence	1256	99	99	*Proteobacteria*
I32.2	*Stenotrophomonas*	−	DQ242478.1	*Stenotrophomonas* sp. D-A 16S ribosomal RNA gene	1361	88	100	*Proteobacteria*
I37.1	*Cellulosimicrobium*	+	AB166888.1	*Cellulosimicrobium* cellulans gene for 16S rRNA, partial sequence	1441	99	99	*Actinobacteria*
I38C	*Cellulosimicrobium*	+	JQ659856.1	*Cellulosimicrobium* funkei strain R6-437 16S ribosomal RNA gene, partial sequence	1472	99	99	*Actinobacteria*
I38D	*Cellulosimicrobium*	+	HM367604.1	*Cellulosimicrobium* sp. GE2 16S ribosomal RNA gene, partial sequence	1375	100	98	*Actinobacteria*
I38E	*Cellulosimicrobium*	+	JQ659856.1	*Cellulosimicrobium* funkei strain R6-437 16S ribosomal RNA gene, partial sequence	1384	100	97	*Actinobacteria*
R7.1	*Agromyces*	+	NR_043931.1	*Agromyces allii* strain UMS-62 16S ribosomal RNA, partial sequence	1365	100	97	*Actinobacteria*
R38.2	*Nocardiopsis*	+	HQ433551.1	*Nocardiopsis* sp. KNU 16S ribosomal RNA gene, partial sequence	1482	99	99	*Actinobacteria*
R38A	*Microbacterium*	+	JQ659823.1	*Microbacterium binotii* strain R6-367 16S ribosomal RNA gene	1411	100	99	*Actinobacteria*
R38-E1	*Microbacterium*	+	JQ659823.1	*Microbacterium binotii* strain R6-367 16S ribosomal RNA gene, partial sequence	1362	99	100	*Actinobacteria*
R40.1	*Klebsiella*	−	JQ305691.1	*Klebsiella variicola* strain ISB-6 16S ribosomal RNA gene, partial sequence	1424	99	99	*Proteobacteria*
R40.2	*Pseudomonas*	−	AB681703.1	*Pseudomonas putida* gene for 16S rRNA, partial sequence, strain: NBRC 102092	1476	99	99	*Proteobacteria*

**Figure 2:**
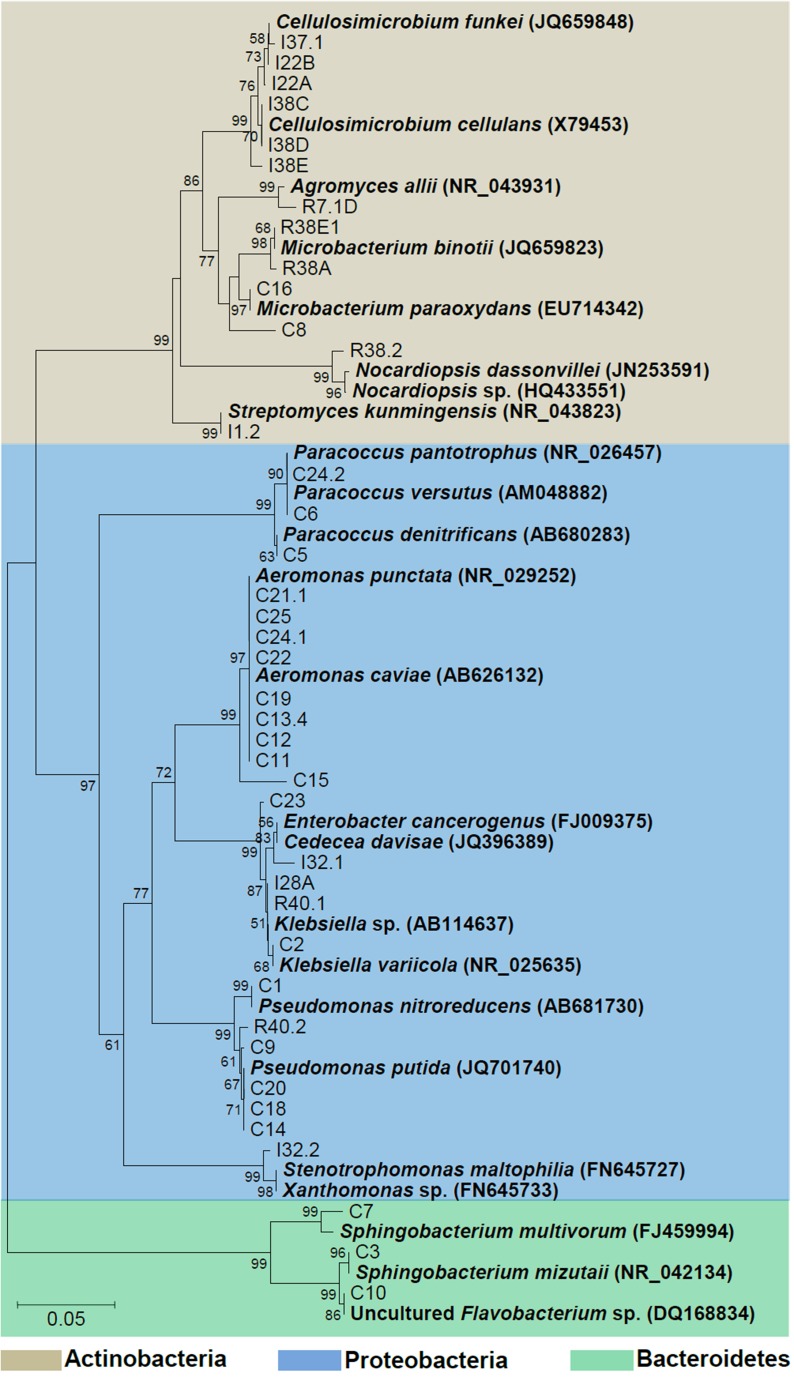
**Phylogenetic tree of isolates**. The 16S sequences of the isolated bacteria are aligned with reference strains. Reference bacterial 16S sequences from GenBank are in bold. Alignments with representative bacterial sequences obtained at GenBank databases were carried out using MUSCLE. Phylogenetic analyses were carried out with MEGA and tree was constructed by neighbor-joining algorithm based on distance estimates calculated by the Kimura-2 parameter which includes a bootstrap test with 1000 replicates. Strains were designated C to indicate isolated from crop; I, from intestine; R, from rectum.

**Figure 3:**
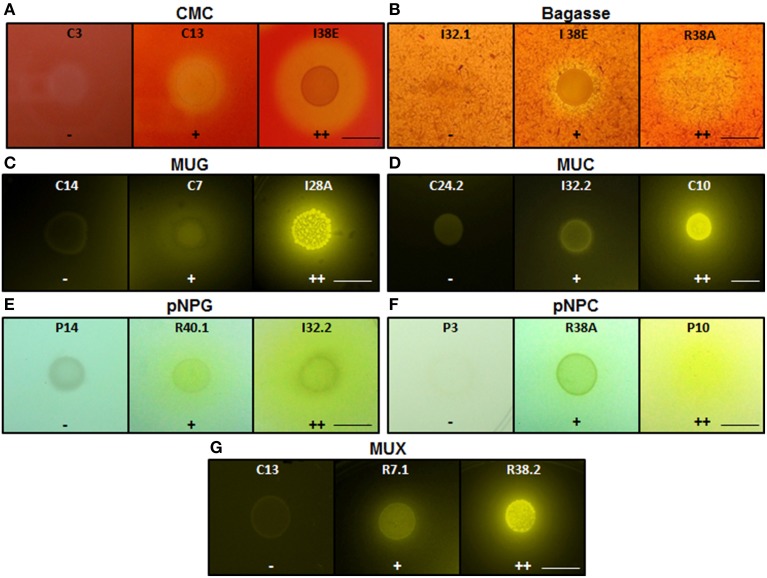
**Enzymatic agar plate assay**. Representative negative, positive, and double positive isolates for each substrate are shown. **(A)** CMC, carboxymethylcellulose; **(B)** Bagasse, powdered sugarcane bagasse; **(C)** MUG, 4-methylumbelliferyl-β-D-glucopyranoside; **(D)** MUC, 4-methylumbelliferyl-β-D-cellobioside; **(E)** pNPG, p-nitrophenyl-β-D-glucopyranoside; **(F)** pNPC, p-nitrophenyl-β-D-cellobioside; and **(G)** MUX, 4-methylumbelliferyl-β-D-xylopyranoside. For bagasse and CMC, the enzyme detection was based on the appearance of negative halo after Congo red stain. For the fluorescent MUC, MUG, and MUX, the plates were UV-irradiated. For the colorimetric substrates pNPC and pNPG, the enzymatic activity was proportional to the development of yellow color. Legends: (−), no detectable hydrolysis; (+), hydrolysis; (++), high hydrolysis. Strains were designated C to indicate isolated from crop; I, from intestine; R, from rectum. Scale bar, 1.0 cm. Note that scale bar applies to all three panels in a series. Also note that strain IDs are shown.

Phylogenetic relationships of the isolates together with representative 16S bacterial sequences were also analyzed (Figure 2). In order to identify the phylogenetic groups that were most efficient in degrading cellulosic compounds, their general repertoire of oligosaccharide-degrading enzymes were evaluated in parallel by enzymatic plate assays (Figure 3). The isolates were ordered by hydrolysis profile similarities and a summary is shown in Table 2. The resulting tree showed that the 40 isolates could be classified into several groups on the basis of similarities in 16S rRNA sequences. Notably, similar hydrolytic profiles could be visualized among phylogenetic-related isolates (Table 2). In the *Cellulosimicrobium* branch, the isolates I22A, I22B, and I37.1 were closely related to *Cellulosimicrobium funkei*, whereas I38C and I38D were more related to *Cellulosimicrobium cellulans* (Figure 2). I38E was put in a separate branch of the tree and showed only 97% of identity with *C. funkei* 16S rRNA sequence (Table 1). Four isolates were grouped in the *Microbacterium* branch. R38A and R38E were closely related to *Microbacterium binotii* (100 and 99% identity, respectively), whereas C8 and C16 were related, in a separate branch and to a lesser extent, to *Microbacterium paraoxydans* (96 and 98% identity, respectively) (Figure 2). This phylogenetic separation between R38A/R38E and C8/C16 agreed well with their cellulolytic potentials. Whereas, R38A and R38E were highly cellulolytic, as showed by the enzymatic plate assay, C8 and C16 were not capable of hydrolyzing the sugarcane bagasse or CMC, only the cello-oligosaccharides MUG, pNPG, and MUC (Table 2). The isolate R7.1 showed 97% identity with *Agromyces allii* strain UMS-62 16S rRNA sequence (NR_043931.1) (Table 1). Although many members of the genus *Agromyces* have been isolated worldwide from soil (Li et al., 2003; Jurado et al., 2005; Yoon et al., 2008; Zhang et al., 2010), their cellulolytic capacities were not reported. The isolates R7.1, R38.2, R38A, R38E, I22A, I22B, I37.1, I38C, I38D and I38E, all from *Actinobacteria* phylum, displayed very similar hydrolytic profiles (Table 2), being able to degrade all of the substrate tested, including the highly recalcitrant powdered sugarcane bagasse. Interestingly, all of the bagasse-degrading isolates also hydrolyzed CMC. All of the CMC- and bagasse-degrading isolates also degrade pNPG and MUG, however, five isolates (C3, C8, C10, C16, I28A, I32.1, I32.2) that secrete β-glucosidase didn't degrade CMC or bagasse.

**Table 2:** **Enzymatic agar plate results ordered by hydrolysis profile**.

**Isolates**	**16S identification**	**CMC**	**Bagasse**	**MUG**	**p-NPG**	**MUC**	**p-NPC**	**MUX**
C8	*Microbacterium*	−	−	+	+	+	−	−
C16	*Microbacterium*	−	−	+	+	+	−	−
R38A	*Microbacterium*	++	++	++	++	++	+	+
R38E	*Microbacterium*	++	++	++	++	++	+	+
I22A	*Cellulosimicrobium*	++	+	++	++	++	+	++
I22B	*Cellulosimicrobium*	++	+	++	++	++	+	++
I37.1	*Cellulosimicrobium*	++	+	++	++	+	+	+
I38C	*Cellulosimicrobium*	++	+	++	++	+	+	++
I38D	*Cellulosimicrobium*	++	+	++	++	+	+	++
I38E	*Cellulosimicrobium*	++	+	++	++	++	+	++
I1.2	*Streptomyces*	++	++	++	++	+	−	−
R7.1	*Agromyces*	+	+	++	++	+	+	+
R38.2	*Nocardiopsis*	+	+	+	+	+	+	+
C11	*Aeromonas*	+	+	++	++	++	+	−
C12	*Aeromonas*	+	++	++	++	+	+	−
C13.4	*Aeromonas*	+	++	++	++	+	+	−
C15	*Aeromonas*	+	++	++	++	+	+	−
C19	*Aeromonas*	+	+	++	+	++	+	−
C21.1	*Aeromonas*	+	+	++	++	+	+	−
C22	*Aeromonas*	+	+	++	++	+	+	−
C24.1	*Aeromonas*	+	−	++	++	++	+	−
C25	*Aeromonas*	+	++	++	++	+	+	−
I32.2	*Stenotrophomonas*	−	−	+	++	+	−	−
C3	*Sphingobacterium*	−	−	+	+	++	−	−
C7	*Sphingobacterium*	+	−	+	+	+	+	−
C10	*Flavobacterium*	−	−	++	++	++	++	++
C2	*Klebsiella*	+	−	++	++	+	+	++
C23	*Klebsiella*	+	−	+	++	+	+	++
I28A	*Klebsiella*	−	−	++	++	+	+	++
I32.1	*Enterobacter*	−	−	+	++	+	−	+
R40.1	*Klebsiella*	+	−	+	+	+	+	+
C1	*Pseudomonas*	−	−	−	−	−	−	−
C5	*Paracoccus*	−	−	−	−	−	−	−
C6	*Paracoccus*	−	−	−	−	−	−	−
C9	*Pseudomonas*	−	−	−	−	−	−	−
C14	*Pseudomonas*	−	−	−	−	−	−	−
C18	*Pseudomonas*	−	−	−	−	−	−	−
C20	*Pseudomonas*	−	−	−	−	−	−	−
C24.2	*Paracoccus*	−	−	−	−	−	+	−
R40.2	*Pseudomonas*	−	−	−	−	−	−	−

*(−) no detectable hydrolysis; (+) hydrolysis; (++) high hydrolysis. Strains were designated C to indicate the ones isolated from crop; I, from intestine; R, from rectum*.

The isolates C11, C12, C13.4, C15, C19, C21.1, C22, C24.1, and C25 were all related to *Aeromonas punctata* and *Aeromonas caviae* (Figure 2) and all of them were capable of hydrolyzing both CMC and the recalcitrant sugarcane bagasse (Table 2), making them promising tools for the discovery of novel cellulases. The isolates C2, C23, I28A, I32.1, and R40.1 were grouped in the *Klebsiella/Enterobacter* branch. Interestingly, these five isolates showed very similar cellulolytic patterns, being able to hydrolyze CMC (except I28 and I32.1), MUG, MUC, and MUX, but not sugarcane bagasse (Table 2). Six isolates were grouped in the *Pseudomonas* branch. C1 was closely related to *Pseudomonas nitroreducens* (99% identity), while C9, C14, C18, C20, and R40.2 were related to *Pseudomonas putida* (98–99% identity) (Figure 2). The isolates C5, C6, and C24.2 were closely related to *Paracoccus denitrificans, Paracoccus versutus*, and *Paracoccus pantotrophus*, with 100, 99, and 99% identity, respectively. All of these isolates showed an identical hydrolytic pattern, none of them being able to secrete detectable amounts of cellulolytic enzymes (Table 2). The isolates C3, C7, and C10 are the only representatives of the phyla *Bacteroidetes* and were related to *Sphingobacterium mizutaii* (99% identity), *Sphingobacterium multivorum* (99% identity), and Uncultured *Flavobacterium* sp. (98% identity) (Figure 2). They are able to hydrolyze pNPG, MUG, pNPC, and MUC, as a consequence for their β-glucosidase secretion, but not CMC or sugarcane bagasse (Table 2). Interestingly, 16 isolates, mainly actinomycetes, hydrolyze the substrate MUX, specific for β-xylosidases (Table 2). β-xylosidases are hydrolytic enzymes which play an important role in xylan degradation, hydrolyzing xylobiose, and xylooligosaccharides from the non-reducing end to xylose. These isolates may be involved in the degradation of hemicellulose, and will be further analyzed in the future for the secretion of other enzymes such as endoxylanases.

### Characterization of bacterial isolates by biochemical tests (Vitek2)

In order to confirm the taxonomic grouping based on 16S rRNA sequences, bacterial isolates were further analyzed by classical biochemical tests using automatized Vitek2 identification System. For preliminary morphological characterization and to confirm the purity of the cultures, the isolates were Gram stained and then visualized under light microscopy and photo-documented (data not shown). Thirteen isolates were found to be Gram positive and 27 Gram negative. Based on the similarities and differences in the biochemical profiles, the isolates could be assigned into six distinct groups among the Gram negative bacteria and into six distinct groups among the Gram positive isolates (Tables 3, 4). As expected, the biochemical profiles obtained from Vitek2 tests agreed well with the 16S taxonomic delimitation (Figure 2), as well with the hydrolytic profile (Table 2).

**Table 3:** **Vitek2 biochemical characterization—GN Card**.

**TEST**	**Mnemonic**	**C1**	**C9**	**C14**	**C18**	**C20**	**R40.2**	**C2**	**C23**	**I28A**	**R40.1**	**I32.1**	**I32.2**	**C3**	**C7**	**C10**	**C5**	**C6**	**C24.2**	**C11**	**C12**	**C13.4**	**C15**	**C19**	**C21**	**C22**	**C24.1**	**C25**
Ala–phe–pro–arylamidase	APPA	−	−	−	−	−	−	−	−	−	−	−	+	+	+	+	−	−	−	+	−	+	+	+	+	+	+	+
Adonitol	ADO	−	−	−	−	−	−	−	−	−	−	−	−	−	−	−	+	+	+	−	−	−	−	−	−	−	−	−
L–pyrrolydonyl–arylamidase	PyrA	−	−	−	−	−	−	−	+	+	+	−	−	−	+	+	−	−	−	−	−	−	−	−	−	−	−	−
L–arabitol	IARL	−	−	−	−	−	−	−	−	−	−	−	−	−	−	−	+	+	+	−	−	−	−	−	−	−	−	−
D–cellobiose	dCEL	−	−	−	−	−	−	+	+	+	+	+	−	−	−	−	−	−	−	+	+	+	+	+	+	+	+	+
Beta–galactosidase	BGAL	−	−	−	−	−	−	+	+	+	+	+	−	−	+	+	−	−	−	+	+	+	+	+	+	+	+	+
H2S production	H2S	−	−	−	−	−	−	−	−	−	−	−	−	−	−	−	−	−	−	−	−	−	−	−	−	−	−	−
B-N-acetylglucosaminidase	BNAG	−	−	−	−	−	−	+	+	−	+	+	−	−	+	+	−	−	−	+	+	+	+	+	+	+	+	+
Glutamyl arylamidase pNA	AGLTp	−	−	−	−	−	−	−	−	−	−	−	−	+	+	+	−	−	−	−	−	−	−	−	−	−	−	−
D–glucose	dGLU	+	+	+	+	+	+	+	+	+	+	+	−	+	−	−	−	−	−	+	+	+	+	+	+	+	+	+
Gama–glutamyl–transferase	GGT	+	+	+	+	+	+	+	+	+	+	+	+	+	−	+	−	−	−	+	−	+	−	−	+	+	−	−
Fermentation/Glucose	OFF	−	−	−	−	−	−	+	+	+	+	+	−	−	−	−	−	−	−	+	+	+	+	+	+	+	+	+
Beta–glucosidase	BGLU	−	−	−	−	−	−	+	+	+	+	+	+	−	+	+	−	−	−	+	+	+	+	+	+	+	+	+
D–maltose	dMAL	+	−	−	−	−	−	+	+	+	+	+	−	−	−	−	−	+	+	+	+	+	+	+	+	+	+	+
D–mannitol	dMAN	−	−	−	−	−	−	+	+	+	+	+	−	−	−	−	+	+	+	+	+	+	+	+	+	+	+	+
D–mannose	dMNE	−	−	+	+	+	+	+	+	+	+	+	−	−	−	−	−	−	−	−	−	−	−	−	−	−	−	−
Beta–xylosidase	BXYL	−	−	−	−	−	−	+	+	+	+	+	−	−	−	+	−	−	−	−	−	−	−	−	−	−	−	−
Beta–alanine acrylamidase	BAlap	+	−	−	−	−	+	−	−	−	−	−	−	+	−	−	−	−	−	−	−	−	−	−	−	−	−	−
L–proline arylamidase	ProA	+	+	+	+	+	+	−	−	+	+	+	+	+	−	−	−	−	−	+	+	+	+	+	+	+	+	+
Lipase	LIP	−	−	−	−	−	−	−	−	−	−	−	+	−	−	−	−	−	−	+	+	+	+	+	+	+	+	+
Palatinose	PLE	−	−	−	−	−	−	+	+	+	+	+	−	−	−	−	−	−	−	−	−	−	−	−	−	−	−	−
Tyrosine arylamidase	TyrA	+	+	+	+	+	+	−	+	+	+	+	−	+	+	+	−	+	+	+	−	+	+	+	+	+	+	+
Urease	URE	+	+	+	+	+	−	−	−	+	+	−	−	−	+	−	−	−	−	−	−	−	−	−	−	−	−	−
D–sorbytol	dSOR	−	−	−	−	−	−	+	+	+	+	+	−	−	−	−	−	+	+	−	−	−	−	−	−	−	−	−
Saccharose/Sucrose	SAC	−	−	−	−	−	−	+	+	+	+	+	−	−	−	−	+	+	+	+	+	+	+	+	+	+	+	+
D–tagatose	dTAG	−	−	−	−	−	−	−	+	−	+	−	−	−	−	−	−	+	−	−	−	−	−	−	−	−	−	−
D–trehalose	dTRE	−	−	−	−	−	−	+	+	+	+	+	−	−	−	−	−	+	+	+	+	+	+	+	+	+	+	+
Citrate (Sodium)	CIT	+	+	+	+	+	+	+	+	+	+	+	+	+	−	−	−	−	−	−	−	−	−	−	−	−	−	−
Malonate	MNT	+	−	+	+	+	−	+	+	+	+	+	+	+	−	−	−	−	−	−	−	−	−	−	−	−	−	−
5-keto-D-gluconate	5KG	−	−	−	−	−	−	+	+	+	+	−	−	−	−	−	−	−	−	−	−	−	−	−	−	−	−	−
L–lactate alkalinisation	ILATk	+	+	+	+	+	+	+	+	+	+	+	+	−	−	−	−	−	−	+	+	+	+	+	+	+	+	+
41 Alpha–glucosidase	AGLU	−	−	−	−	−	−	−	−	−	−	−	−	−	+	+	−	−	−	−	−	−	−	−	−	−	−	−
Succinate alkalinisation	SUCT	+	+	+	+	+	+	+	+	+	+	+	+	+	−	−	−	+	−	+	+	+	+	+	+	+	+	+
B-N-acetylgalactosaminidase	NAGA	−	−	−	−	−	−	−	−	−	−	+	−	−	+	+	−	−	−	−	−	−	−	−	−	−	−	−
Alpha–galactosidase	AGAL	−	−	−	−	−	−	+	+	+	+	+	−	−	+	+	−	−	−	−	−	−	−	−	−	−	−	−
Phosphatase	PHOS	+	−	−	−	−	−	+	+	+	+	+	+	+	+	+	−	−	−	−	−	−	−	−	−	−	−	−
Glycine arylamidase	GlyA	+	−	−	−	−	−	−	−	+	+	+	−	−	−	+	−	−	−	−	−	+	−	−	−	+	−	−
Ornithine decarboxylase	ODC	−	−	−	−	−	−	+	−	−	−	+	−	−	−	−	−	−	−	−	−	−	−	−	−	−	−	−
Lysine decarboxylase	LDC	−	−	−	−	−	−	+	+	+	+	−	−	−	−	−	−	−	−	−	−	−	−	−	−	−	−	−
L–histidine assimilation	IHISa	+	+	+	+	+	+	−	−	−	−	−	−	−	−	−	−	−	−	−	−	−	−	−	−	−	−	−
Coumarate	CMT	+	+	+	+	+	+	−	−	+	−	−	−	−	−	−	−	−	−	+	+	+	+	+	+	+	+	+
Beta–glucuronidase	BGUR	−	−	−	−	−	−	−	−	−	−	−	−	−	−	−	−	−	−	−	−	−	−	−	−	−	−	−
O/129 resistance	O129R	+	+	+	+	+	+	+	+	+	+	+	−	+	−	−	−	−	−	+	+	+	+	+	+	+	+	+
Glu–gly–arg–arylamidase	GGAA	−	−	−	−	−	−	−	−	−	−	−	+	−	+	+	−	−	−	+	−	+	−	+	+	+	−	−
L–malate assimilation	IMLTa	+	+	+	+	+	−	−	−	−	−	−	−	+	−	−	−	+	+	−	−	+	+	+	+	+	+	+
Ellman	ELLM	−	−	−	−	−	−	−	−	−	−	−	−	−	−	+	+	+	+	+	+	+	+	+	+	+	+	+
L–lactate assimilation	ILATa	+	+	+	+	+	−	−	−	−	−	−	−	+	−	−	−	−	−	−	−	−	−	−	+	+	−	−

**Table 4:** **Vitek 2 biochemical characterization—GP Card**.

**TEST**	**Mnemonic**	**C8**	**C16**	**R38A**	**R38E**	**I22A**	**I22B**	**I38C**	**I38D**	**I38E**	**I37.1**	**I1.2**	**R7.1**	**R38.2**
D-amygdalin	AMY	−	−	−	−	−	−	−	−	−	−	−	−	+
Phosphatidylinositolphospholip	PIPLC	−	−	−	−	−	−	−	−	−	−	−	−	−
D-xylose	dXYL	−	−	+	+	+	+	+	+	+	+	−	+	−
Arginine dihydrolase	ADH1	+	+	+	+	+	−	−	−	−	−	−	+	−
Beta-galactosidase	BGAL	+	+	+	+	−	−	+	+	+	+	−	+	−
Alfa-glucosidase	AGLU	+	+	+	+	+	+	+	+	+	+	+	+	+
Ala-phe-pro arylamidase	APPA	+	+	+	+	+	+	+	+	+	+	+	−	+
Cyclodextrin	CDEX	−	−	−	−	−	−	−	−	−	−	−	−	−
L-aspartate arylamidase	AspA	−	−	−	−	−	−	−	−	−	−	−	−	−
Beta galactopyranosidase	BGAR	−	−	−	−	−	−	+	+	+	−	−	+	−
Alfa-mannosidase	AMAN	+	+	−	−	−	−	−	−	−	−	−	−	−
Alkaline phosphatase	PHOS	−	−	−	−	−	−	−	−	−	−	−	−	−
Leucine arylamidase	LeuA	+	+	+	+	+	+	+	+	+	+	+	+	+
L-Proline arylamidase	ProA	+	+	+	+	+	+	+	+	+	+	+	+	+
Beta glucuronidase	BGURr	−	−	−	−	−	−	−	−	−	−	−	−	+
Alpha-galactosidase	AGAL	−	−	+	+	−	−	−	−	−	−	−	−	+
L-Pyrrolidonyl-arylamidase	PyrA	−	−	−	−	+	+	+	+	+	+	−	−	−
Beta glucuronidase	BGUR	−	−	−	−	−	−	−	−	−	−	−	−	−
Alanine arylamidase	ALaA	+	+	+	+	+	+	+	+	+	+	+	+	+
Tyrosine arylamidase	TyrA	+	+	+	+	+	+	+	+	+	+	−	+	+
D-sorbitol	dSOR	−	−	−	−	−	−	−	−	−	−	−	−	−
Urease	URE	−	−	−	−	−	−	−	−	−	−	+	−	+
Polymixin B resistance	POLYB	−	−	−	−	−	−	−	−	−	−	−	−	−
D-galactose	dGAL	+	+	+	+	+	+	+	+	+	+	+	+	−
D-RiBOSE	dRIB	−	+	−	−	−	−	+	+	+	+	−	−	−
L-lactate alkalinisation	ILATk	+	+	+	+	−	−	+	+	+	−	−	−	−
Lactose	LAC	−	−	−	−	−	−	−	−	−	−	−	−	−
N-acetyl-D-glucosamine	NAG	−	−	−	−	+	+	+	+	+	+	−	−	−
D-maltose	dMAL	+	+	+	+	+	+	+	+	+	+	−	+	−
Bacitracin resistance	BACI	−	+	−	−	−	−	−	−	−	−	−	−	−
Novobiocin resistance	NOVO	−	−	−	−	−	−	+	+	+	−	−	−	−
Growth in 6.5% NaCl	NC6.5	−	−	−	−	−	−	−	−	−	−	−	−	−
D-mannitol	dMAN	+	+	+	+	−	−	−	−	−	−	−	−	−
D-mannose	dMNE	+	+	+	+	+	+	+	+	+	+	−	+	−
Methyl-B-D-glucopyranoside	MBdG	−	−	+	+	−	−	−	−	−	−	−	−	−
Pullulan	PUL	−	−	−	−	−	−	−	−	−	−	−	−	−
D-raffinose	dRAF	−	−	−	−	−	−	−	−	−	−	−	−	−
O/129 resistance (comp. Vibrio.)	O129R	−	−	−	−	−	−	−	+	+	−	−	−	−
Salicin	SAL	−	−	+	+	−	−	−	−	+	−	−	+	−
Saccharose/Sucrose	SAC	+	+	+	+	+	+	+	+	+	+	+	−	−
D-treahlose	dTRE	+	+	+	+	+	+	+	+	+	+	−	−	−
Arginine dihydrolase 2	ADH2s	−	−	−	−	−	−	−	−	−	−	−	−	+
Optochin resistance	OPTO	−	−	+	+	+	+	+	+	+	+	−	−	−

The Gram negative isolates C1, C9, C14, C18, C20, and R40.2 (*Pseudomonas* group, Figure 2) shared several biochemical traits that confirm they are all taxonomically related (Table 3, Figure 2). However, three bacterial isolates showed slight differences from each other in such biochemical properties as D-maltose utilization, presence of phosphatase and glycine arylamidase (C1 only), presence of beta-Alanine acrylamidase (C1 and R40.2), absence of urease (R40.2 only), inability to assimilate L-malate and L-lactate (R40.2), lack of malonate acidification (C9 and R40.2), and inability to utilize D-mannose (C1 and C9).

The second discernible group is represented by the isolates C2, C23, I28A, I32.1, and R40.1 (*Klebsiella* group, Figure 2). Two isolates showed slight differences, such as presence of L-pyrrolydonyl-arylamidase (C23) and absence of tyrosine arylamidase and ornithine decarboxylase absence (C2). The isolate I32.2 displayed a biochemical pattern that was distinct from all the others, which confirms it is the unique representative of its taxonomic group, *Stenotrophomonas* (Figure 2). The two following groups (the one represented by C3 and C7 and the other by C5, C6, C24.2, respectively) were the most heterogeneous. This suggests that each of them constitutes distinct species from *Sphingobacterium* and *Paracoccus* genus, respectively. The last discernible group among Gram negative isolates contains C11, C12, C13.4, C15, C19, C21, C22, C24.1, and C25 (*Aeromonas* group, Figure 2). All were able to utilize D-cellobiose, to ferment glucose and were positive for the beta-glucosidase test (Table 3). Despite the overall similarities in the biochemical profile, only the isolates C24.1 and C25 were identical. The others showed slight differences, such as absence of the enzymes Ala-Phe-Pro-Arylamidase and Tyr-arylamidase (C12), presence of glycine arylamidase (C13.4 and C22), inability to assimilate L-malate (C11 and C22), and L-Lactate assimilation (C21 and C22). These slight biochemical differences suggest genetic diversity in the species level among these phenotypically related isolates.

The Gram positives isolates C8 and C16 displayed very similar biochemical profiles (Table 4), differing only in the D-ribose utilization and bacitracin resistance (C16 positive) and were closely related to *M. paraoxydans* (Figure 2). The isolates R38A and R38E displayed identical profiles and were closed related to *M. binotii* (Table 4, Figure 2). The third discernible group among Gram positives is represented by the isolates I22A, I22B, I38C, I38D, I38E, and I37.1 (*Cellulosimicrobium* group, Figure 2). Although I22A, I22B, and I37.1 showed very similar profiles, they are not identical, differing in the arginine dihydrolase trait (only I22A was positive), in D-ribose utilization, and presence of beta-galactosidase (I37.1 only). Likewise, the closely related I38C, I38D, and I38E, differ from I22 by the presence of beta-galactopiranosidase, L-lactate alkalinisation, and novobiocin resistance. Differences in these three isolates, however, occur in the Salicin and O/129 resistance (Table 4). Finally, each of the isolates I1.2, R7.1, and R38.2 displayed an unique biochemical pattern, which confirms they are the sole representatives of their taxonomic group *Streptomyces, Agromyces*, and *Nocardiopsis*, respectively (Figure 2).

## Discussion

The land snail *A. fulica* is a voracious herbivorous with great environmental and ecological importance. Most of its capacity to process a broad variety of vegetable organic matter is due to the presence of cellulolytic enzymes, both from the animal and resident microbiota. The bacterial communities inside the gut of this snail may have crucial importance in cellulose and other plant wall components digestion. As the first steps of this study, we assumed that the different gut regions such as the crop, intestine and rectum are highly specialized compartments, and each could have a distinct role to play in digestion, as well as particular resident microbial communities. Based on the 16S rRNA gene sequence, our 40 isolates showed their closest matches to 13 distinct genera, six of the *Proteobacteria* phyla (*Aeromonas, Pseudomonas, Klebsiella, Enterobacte*r, *Stenotrophomonas*, and *Paracoccus*), five of *Actinobacteria* (*Streptomyces, Cellulosimicrobium, Agromyces, Microbacterium*, and *Nocardiopsis*), and two of *Bacteroidetes* (*Sphingobacterium* and *Flavobacterium*). Although we have selected exclusively cultivable CMC-degrading bacterial species in our screening method, many of the genera identified in this work were reported in previous studies based on metagenomic approaches (Cardoso et al., 2012a,b), which per definition detect also non-cultivable, anaerobic, and non-cellulolytic species. For instance, Cardoso et al. (2012a) identified the following bacterial taxa whose representatives were also isolated in our study: *Enterobacter* (24 clones) *Klebsiella* (16 clones), *Aeromonas* (89 clones, 87 from crop fluid), *Pseudomonas* (38 clones), *Xanthomonas* (48 clones), *Microbacterium* (4 clones, exclusively from rectum), and *Flavobacterium* (25 clones). Similar to our findings, Cardoso et al. (2012a) showed that the bacterial community structure of crop fluid was different from that of the feces (named rectum, in our study), suggesting that this land snail microbiota changes according to the gut region. Besides the above mentioned species, they were able to detect representative 16S rRNA sequences of the following taxa, which were not isolated in our study: *Sulfurospirillum* (72 clones), *Citrobacter* (39 clones) *Clostridiaceae* (47 clones), *Lactococcus* (44 clones), and *Mucilaginibacter* (70 clones). The reasons that could account for the absence of these taxa in our screening is the need for anaerobic or microaerophilic conditions for growth, in the case of *Clostridiaceae* (Ko et al., 2011) and *Sulfurospirillum* (Lancaster and Simon, 2002; Luijten et al., 2003); the inability to degrade CMC, in the case of non-cellulolytic species of the *Sulfurospirilum* genus (Pankratov et al., 2007), and the natural shift in gut microbial communities that takes place in snails according to their diet (Cardoso et al., 2012a). Likewise, in an independent metagenomic analysis of the microbiota from the crop of *A. fulica* (Cardoso et al., 2012b), the genera *Pseudomonas* (37.5%), *Sulfurospirillum* (8.5%), and *Stenotrophomonas* (7.3%) were assigned as principal bacterial groups.

The 16S rRNA based taxonomic delimitation was corroborated by our biochemical profiling using the Vitek cards. Based on similarities and differences on the biochemical profile, the isolates could be assigned into 12 distinct groups (Tables 3, 4) whose component isolates are grouped in a very similar way in the phylogenetic tree (Figure 2). Besides, it is noteworthy that all of these phenotypic-related isolates belong to the same cellulolytic profile group (Table 2), indicating a clear correlation between molecular taxonomy, biochemical profile, and cellulolytic phenotype.

The isolates C14, C18, and C20 exhibited the same biochemical Vitek profile and were placed in the same cluster (together with *P. putida*) in the 16S rRNA gene tree, with zero distance (Figure 2). This which may indicate that these isolates could be multiples of the same organism. By the other hand, although isolates C11, C12, C13.4, C19, C21.1, C22, C24.1, and C25 have been placed in the same cluster in the phylogenetic tree with zero distance, it is not sufficient to confirm that they are the same organism. Firstly because two distinct representative type strains (*A. punctata* and *A. caviae*) were placed together with these isolates, suggesting that 16S rRNA sequence alone could not provide enough taxonomic discriminatory power. Secondly, recent work has shown that multilocus phylogenetic analysis (MLPA) of at least five concatenated housekeeping genes is a more accurate tool for the delineation of *Aeromonas* species (Martinez-Murcia et al., 2011). Housekeeping genes evolve faster than the 16S rRNA, have a higher resolution for differentiating closely related species and therefore are more reliable for the correct identification of *Aeromonas* strains to species level. Finally, it can be seen from Table 3 that only isolates C24.1 and C25 shared the same biochemical Vitek profile, while the closely related C11, C12, C13.4, C19, C21.1, and C22 isolates display some differences regarding presence of Ala–Phe–Pro–arylamidase, gama–glutamyl–transferase, tyrosine and glycine arylamidase, Glu–Gly–Arg– arylamidase, and L–lactate assimilation. In the case of the *Aeromonas* strains recovered in this study, the extent of clonal duplication in the isolate pools remains undetermined and additional studies using MLPA or genome sequencing will be necessary to identify which isolates are multiples of the same organism.

In this study, the bacterial isolates that displayed the greatest cellulolytic potential (Table 2) belong to the *Actinobacteria* phylum: R38A and R38E (*Microbacterium* species), I22A, I22B, I38C, I38D, I38E, and I37.1 (*Cellulosimicrobium* species), I1.2 (*Streptomyces* sp.); R7.1 (*Agromyces* sp.), and R38.2 (*Nocardiopsis* sp.). *Actinomycetes*, which are Gram positive filamentous bacteria, are well known for their ability to decompose complex molecules, particularly the lignocellulose components, which make them important agents in decomposition processes (Lacey, 1997). According to Pawar et al. (2012), very few (< 1%) sequences in esophagus, crop, stomach, and rectum libraries were related to *Actinobacteria*, and in an intestine library they were completely absent. Cardoso et al. (2012a) showed that *Actinobacteria* were the minority phyla both inside the crop and the rectum (feces), but the intestinal bacterial community was not evaluated. Since the majority of our *Actinobacteria* isolates were obtained from the intestine lumen, this could explain the low percentage obtained in this previous work. Regardless, our results show that *Actinobacteria* representatives could be easily recovered from the intestinal tract of *A. fulica* and cultivated in order to produce a wide range of glycoside hydrolases. Although many members of the genus *Agromyces* have been isolated worldwide from soil (Li et al., 2003; Jurado et al., 2005; Yoon et al., 2008; Zhang et al., 2010), their cellulolytic capacities were not previously reported. Nevertheless, our enzymatic plate assay results showed that R7.1 degraded all the substrates tested, suggesting that *Agromyces* species can be valuable candidates for cellulase and xylanase production (Table 2). Interestingly, all of the *Aeromonas* isolates were capable of hydrolyzing CMC and the recalcitrant sugarcane bagasse, making them promising tools for the discovery of novel cellulases. This genus, unlike the relatively well characterized cellulolytic *Cellulosimicrobium* (Bakalidou et al., 2002; Kim do et al., 2012) and *Streptomyces* (Garda et al., 1997; Da Vinha et al., 2011), has been so far underestimated as a source of cellulolytic species. Our results show that the snail *A. fulica* is a source of distinct *Aeromonas* species that are very promising for cellulase production (Tables 2, 3 and Figure 2). Among 40 cellulolytic isolates retrieved in our study, a total of 10 isolates (I22A, I22B, I37.1, I38C, I38D, I38E, R7.1, R38.2, R38A, R38E) were able to hydrolyze all of the substrates tested in our plate assay, including the recalcitrant sugarcane bagasse, even without its pre-treatment (Table 2). This suggests that these isolates are able to secrete a bulk of lignocellulolytic enzymes that breaks the complex structure of the sugarcane cell wall. This enzymatic bulk may include endoglucanases, cellobiohydrolases, and β-glucosidases for the hydrolysis of the cellulosic cell wall component; while other enzymes, such as endoxylanases and β-xylosidases could account for the hydrolysis of the hemicellulose fraction.

Based on a culture-dependent CMC-degrading bacteria screening method, this study for the first time demonstrates that the cellulolytic flora in the gastrointestinal tract of *A. fulica* can be easily recovered in order to produce several hydrolytic enzymes. Besides, this diversity changes according to the gut segment: while in the crop the proteobacteria *Aeromonas* was predominant, in the intestine the well characterized *Actinobacteria* phylum harbored the majority of the isolates, mainly *Cellulosimicrobium* genus representatives. This study extends the current knowledge of the *A. fulica* microbiota, and is the first investigation that specifically recovers cellulolytic bacteria from *A. fulica* by culture-dependent methods, making possible to use these isolates in fermentation processes for enzyme production or as source of novel genes for heterologous protein expression. Preliminary results of this study indicate that isolated bacteria are able to produce a diversity of enzymes and can degrade the highly recalcitrant sugarcane bagasse. Our future work will include the detailed genomic, biochemical and proteomic characterization of the secretome from selected isolates, in order to evaluate their lignocellulose-degrading potential on biotechnological processes.

### Conflict of interest statement

The authors declare that the research was conducted in the absence of any commercial or financial relationships that could be construed as a potential conflict of interest.
