# Supplementary material for: Isolation of aerobic cultivable cellulolytic bacteria from different regions of the gastrointestinal tract of giant land snail Achatina fulica
Source: Front Microbiol. 2015 Aug 20;6:860. doi: 10.3389/fmicb.2015.00860 (PMC4542579; doi:10.3389/fmicb.2015.00860)
Supplement: Supplementary file 1 [file Table1.DOCX]

**Table 1S.- Accession numbers (Supplemental)**

| Isolate | Accession Number |
| --- | --- |
| C1 | KF530754 |
| C2 | KF530755 |
| C3 | KF530756 |
| C5 | KF530757 |
| C6 | KF530758 |
| C7 | KF530759 |
| C8 | KF530760 |
| C9 | KF530761 |
| C10 | KF530762 |
| C11 | KF530763 |
| C12 | KF530764 |
| C13.4 | KF530765 |
| C14 | KF530766 |
| C15 | KF530767 |
| C16 | KF530768 |
| C18 | KF530769 |
| C19 | KF530770 |
| C20 | KF530771 |
| C21.1 | KF530772 |
| C22 | KF530773 |
| C23 | KF530774 |
| C24.1 | KF530775 |
| C24.2 | KF530776 |
| C25 | KF530777 |
| I 1.2 | KF530778 |
| I22A | KF530779 |
| I22B | KF530780 |
| I28A | KF530781 |
| I32.1 | KF530782 |
| I32.2 | KF530783 |
| I37.1 | KF530784 |
| I38C | KF530785 |
| I38D | KF530786 |
| I38E | KF530787 |
| R7.1 | KF530788 |
| R38.2 | KF530789 |
| R38A | KF530790 |
| R38-E1 | KF530791 |
| R40.1 | KF530792 |
| R40.2 | KF530793 |
